# Exploring the Effectiveness of Practice Development Interventions on Patient and Staff Outcomes: A Systematic Review

**DOI:** 10.1111/jan.70661

**Published:** 2026-07-04

**Authors:** Hazel A. Smith, Vanda Carter, Charlotte Phillips, Yetunde Ataiyero, Alisen Dube, Joanne Odell, Callum Latham, Sally Hardy, Alison Leary, Sarahjane Jones

**Affiliations:** ^1^ Centre for Health Innovation/Centre for NMAHP Research and Education Excellence (CeNREE) University of Staffordshire/University Hospitals of North Midlands NHS Trust Stoke‐on‐Trent UK; ^2^ Centre for Health Innovation University of Staffordshire Stoke‐on‐Trent UK; ^3^ Norfolk Initiative for Coastal and Rural Health Equalities (NICHE), University of East Anglia Norwich UK; ^4^ London South Bank University London UK

**Keywords:** participatory action research, patient outcomes, practice development, staff outcomes, systematic review

## Abstract

**Aim:**

To explore Practice Development interventions and associated contextual factors to understand their impact on patient and staff outcomes and experiences in healthcare settings.

**Design:**

Systematic review.

**Methods:**

Preferred Reporting Items for Systematic Reviews and Meta‐analysis informed design, conduct and management. A systematic approach was taken, utilising multiple reviewers and consensus methods. Mixed Methods Appraisal Tool informed quality appraisal. Qualitative findings were analysed using Reflexive Thematic Analysis. Quantitative results are presented as a narrative summary.

**Data Sources:**

CINAHL; MEDLINE; PubMed; Scopus; ScienceDirect; Nursing and Allied Health; Cochrane Library; Internurse.com; The Kings Fund; GOV.UK and Trip Database were searched between 1st January 1980 to 5th January 2026.

**Results:**

Twenty‐nine studies were included. Eighteen studies met all quality assessment criteria. Limited quantitative data was available, and inconsistent outcome measures constrained quantitative analysis. Qualitative analysis generated three themes: (1) Shaping Practice: the interplay of organisational context, culture, and structure, (2) Enablers and constraints in Practice Development, (3) Collective empowerment.

**Conclusion:**

Across diverse study designs, the majority of studies indicate that PD contributes positively to staff working environments and the contexts in which patient care is delivered. However, current findings do not demonstrate consistent improvements in clinical outcomes or quality of life.

**Implications for Practice:**

Practice Development may contribute to improvements in care environments for patients and staff, respectively. This review highlights knowledge gaps in the generalisability and transferability of Practice Development that would benefit from further exploration.

**Impact:**

Practice Development may enhance healthcare environments, but further research is needed to determine if these benefits impact upon clinical outcomes.

**Reporting Method:**

Preferred Reporting Items for Systematic reviews and Meta‐Analyses.

**No Patient or Public Involvement:**

No patient or public contribution.

**Trial Registration:**

PROSPERO registration number: CRD42024557949

## 1 Introduction

Healthcare operates in a continuum of change, reform, modernisation, and transformation. One method of promoting organisational change, clinical governance, clinical effectiveness, and person‐centred care includes Practice Development (PD) (Manley et al. 2014). PD originated in the 1980s through the establishment of nursing development units (NDUs), which supported nurses to promote care standards and quality improvement systems. These units evolved into practice development units as they expanded the focus to multidisciplinary collaboration and improved patient outcomes (Bradd et al. 2017). Today, PD constitutes a complex, emancipatory methodology designed to advance person‐centred, evidence‐informed healthcare. It positions practitioners as active agents in transformative processes, employing iterative, action‐oriented and evaluative cycles to interrogate and refine evidence, thereby embedding and sustaining innovations that promote safety and effectiveness. Central to PD is an emphasis on person‐centred care, organisational culture, and systemic dynamics, alongside an explicit commitment to engaging with complexity and research practice conducted with individuals rather than on them (Manley et al. 2014).

PD is widely used in nursing in the United Kingdom (UK) and is recognised as a structured and rigorous process, supported by facilitation methods, that focuses on enhancing the quality of nursing care by transforming care practices and cultures (Garbett and McCormack 2002). PD represents a dynamic approach and progressive force in the evolution of nursing practice, healthcare delivery, and organisational change (McCormack and McCance 2017). In addition, PD enables health and social care workers, teams, and organisations to transform the culture and context in which care is provided (McSherry and Warr 2008). PD is a multifaceted intervention methodology rooted in an emancipatory approach, aiming to guide person‐centred evidence‐based healthcare (Manley et al. 2011; Manley et al. 2021). It represents an ongoing commitment to improving healthcare systems, transcending intervention changes to necessitate a cultural shift in care delivery (McCormack et al. 1999). This evolution reflects a transition from traditional, task‐focused nursing models towards individualised, person‐centred approaches, driven by the need to counter the dehumanising effects of routine‐oriented care within hospital systems. PD involves working with practitioners by engaging them as active participants of change, using action‐oriented and evaluation cycles that continue to inform and critique the evidence to sustain new initiatives, for safe and effective practices (Manley et al. 2011). Moreover, PD encompasses a mechanism for contemplation of day‐to‐day practice, empowering care providers to institute modifications that promote improved clinical outcomes and elevate care quality and safety.

PD impacts individuals, teams, user experiences, and practitioners. However, it is argued that PD's potential to influence broader systems remains underutilised (Manley et al. 2021). Managerial task‐based cultures or single top‐down methodology approaches often ignore the importance of engaging with people or fail to use practitioners' expertise as a source of social capital. The emphasis for PD remains on person‐centred care, cultures, and systems as well as working with complexity and research practice “with people” rather than “on” (Manley et al. 2021).

Participatory Action Research (PAR) has been found to be an enabler for practice change in nursing (Buckley et al. 2022). PAR is a research‐to‐action methodological approach and is a sub‐category of action research. PAR is rooted in social psychology (Cusack et al. 2018; Polit‐O'Hara and Beck 2008) and prompts people to recognise what needs changing and how change can happen (University of Reading 2023). Instead of observing reality, PAR encourages collaboration and solidarity to drive change. Through PAR, questioning long held beliefs and practice contributes to knowledge generation (Cusack et al. 2018). By shedding light on traditional hierarchies and promoting emancipatory action, PAR initiates change. PAR involves a cyclical process: first “looking” (collecting evidence about a situation), then “thinking” (reflecting critically on the evidence together), and finally “acting” (developing a shared action plan for action). Beyond describing reality, PAR strives for social justice through emancipatory change potentially leading to sustainable transformation (Odell 2024). PAR aims to foster an equitable, diverse, and inclusive research process (Lenette 2022) where active participation brings insights and valuable lessons of self‐regulation, personal and professional advancement (Groot and Abma 2022).

The primary aim of this review is to explore the existing literature on PD interventions, with and without PAR, involving patient and/or staff and reporting on outcomes and/or experiences. Specifically, we aim to analyse the contextual factors associated with PD intervention and their impact on patient and/or staff outcomes and/or experiences, while also investigating the relationship between PD intervention characteristics and patient and/or staff outcomes and/or experiences.

## 2 Methods

### 2.1 Design

The Preferred Reporting Items for Systematic Reviews and Meta‐analysis Protocols (Page et al. 2021) was used for the design, conduct and management of this study. The protocol for this systematic review and mixed‐methods analysis is registered with the International Prospective Register of Systematic Reviews (PROSPERO), registration number CRD42024557949 (Smith et al. 2024). This mixed methods review is underpinned by a pragmatic philosophy. Pragmatism's focus on actionable knowledge is noted to be a useful, action‐oriented approach to research concerning organisational processes and can ensure that research is contextually relevant (Kelly and Cordeiro 2020). For this reason, pragmatism was selected as it was deemed congruent with the principles of PD.

### 2.2 Search Strategies

The following databases were searched: CINAHL; MEDLINE; PubMed; Scopus; ScienceDirect; Nursing and Allied Health; Cochrane Library; Internurse.com; The Kings Fund; GOV.UK; and Trip Database.

For the majority of searches, the same strategy was utilised and is listed in Table 1. The Kings Fund, Science Direct and Internurse required adaptations to be made to the search strategy. For Kings Fund, a full search could not be performed, so “Practice Development” and “Participatory Action Research” were searched separately. Science Direct was limited to eight Boolean operators and Internurse had records available from January 1992.

**TABLE 1:** Search strategy.

Search step	Description
Line 1 (Search 1: Population)	“Staff” OR “Healthcare Prof*” OR “Nurs*” OR “Clinical” OR “Medical” OR “Allied Health” OR “Art Therap*” OR “Dietiti*” OR “Music Therap*” OR “Operating Department Practit*” OR “Orthopt*” OR “Osteop*” OR “Paramed*” OR “Physiotherap*” OR “Podiatr*” OR “Prosthet*” OR “Orthos*” OR “Radiograph*” OR “Speech and Language Therap*” OR “Patient” OR “Service User” OR “Client” OR “Consumer” OR “Care Recipient*”
Line 2 (Search 2: Intervention)	Proximity Search Input** (“Practice Development”)
Line 3 (Search 3: Intervention)	Proximity Search Input ***(“Participatory Action Research”)
Line 4 (Search 4)	Search 2 OR Search 3
Line 5 (Search 5)	Combine Search 1 AND Search 4
Line 6 (Apply Limiters)	1st January 1980 until 5th January 2026

### 2.3 Eligibility Criteria

Primary research, with no restrictions on study methodology, published between 1st January 1980 up to 5th January 2026 was included. Studies were required to have been conducted in a healthcare setting and must have included (1) methods of implementing the PD intervention and (2) at least one patient and/or staff outcome and/or experience on impact of PD intervention with or without PAR.

Two limitations were applied: language and setting. Only papers published in the English language were included. Maternity‐specific settings were excluded as this setting is not relevant to the wider project aims. Neonatal nursing settings were included.

Although the term ‘Practice Development’ is widely used it is utilised inconsistently across education, research and audit (Garbett and McCormack 2002). Therefore, the review team acknowledge that the term ‘Practice Development’ may not be used to reflect the same process of work in all countries. Hardy et al.'s (2021) (Hardy et al. 2021) principles for PD (Table 2) were utilised to underpin definitions and assumptions concerning PD. PAR is a cyclical process of defining the problem, designing appropriate actions, observing and gathering data on targeted outcomes, analysing and reflecting on the results and then repeating this process as often as is needed (Cornish et al. 2023).

**TABLE 2:** Defining practice development.

Category	Description
PD foundations	PD is fundamentally about person‐centred practice that promotes safe and effective workplace cultures where all can flourish. PD uses collaborative inclusive participatory approaches
Processes	PD blends creativity with learning, freeing individuals' hearts, minds, and souls to achieve new ways of thinking, doing, and being. PD utilises active work‐based learning to facilitate individual, practice, and cultural transformation. PD is a facilitated process that seeks to promote critical informed action. PD uses inclusive evaluation to integrate evidence from processes and outcomes of transformation
Outcomes	PD focuses on supportive relationships across individuals, teams, and systems to stimulate effective change. PD is a complex methodology that uses a variety of evidence to inform transformation for individuals, teams, and systems

*Source:* Hardy et al. (2021).

### 2.4 Screening Process

Covidence, a web‐based software that assists researchers in screening references and undertaking data extraction, was used for all stages of screening and data extraction. Three reviewers (HAS, CL and VC) independently reviewed the titles and abstracts of the retrieved records to evaluate eligibility. HAS screened all papers; CL and VC shared the role of reviewer. Any discrepancies were discussed and resolved, with a fourth reviewer (JO) available as required. If uncertainty remained, the paper was moved to full review for further examination. Papers were also moved to full review if the members of the research team felt there was insufficient information in the title or abstract to make an informed decision on the paper's eligibility.

This process was again followed for full paper review for inclusion. Study authors were also contacted to resolve any outstanding queries or to enable access to papers.

### 2.5 Data Extraction

A data extraction template mapped to the PICO (Participants, Intervention, Comparator, and Outcome) framework was generated by the review team. The data extraction template included but was not limited to: PD and PAR Definitions and Characteristics, Contextual Factors (organisational culture and structure, leadership, resources, staff engagement, collaboration and communication, patient involvement, training and education, evaluation and feedback), Patient Outcomes (clinical outcomes, patient experiences, quality of life, patient satisfaction) and Staff Outcomes (facilitators and barriers to care delivery, job satisfaction, experiences of care delivery, burnout, workforce engagement, staff wellbeing, and team dynamics).

Two members of the study team (HAS and VC) independently undertook all data extraction. A third reviewer (CP) further reviewed the two sets of extracted data for alignment and completeness before merging the extracted data. Any discrepancies or queries were resolved by a fourth reviewer (JO).

### 2.6 Risk of Bias Assessment

Two members of the study team (HAS and VC) independently critical appraised each study using the Mixed Methods Appraisal Tool (MMAT) Version 2018 for mixed‐method studies (Hong et al. 2018). Any discrepancies were resolved by consensus. MMAT is a single validated tool for mixed methods systematic reviews to enable consistent quality assessment across the most common types of methodologies (qualitative, quantitative randomised controlled trails, quantitative non‐randomised, quantitative descriptive and mixed methods). The focus of MMAT is on methodological quality and not on the quality of reporting (Hong et al. 2018). The MMAT emphasises descriptive reporting and does not recommend calculating an overall score. Instead, each criterion is assessed as ‘met’, ‘not met’, or ‘can't tell’. MMAT did not form any part of the screening process, and no studies were excluded based on methodological quality alone.

### 2.7 Data Synthesis

PD is iteratively tailored to the needs of the staff, patients, carers, and organisations. Therefore, the ability to conduct a meta‐analysis was not anticipated due to the heterogeneity inherent in PD methodology. This was confirmed at the quantitative data analysis stage. Qualitative data was analysed and presented separately.

#### 2.7.1 Quantitative Data

Study author (HAS) conducted the quantitative analysis to present a narrative summary of the findings from the included data.

#### 2.7.2 Qualitative Data

Findings from the qualitative studies were analysed using reflexive thematic analysis, following Braun & Clarke's six defined steps (Braun and Clarke 2019); familiarisation with the data, generating initial codes, constructing themes, reviewing potential themes, defining and naming themes, and producing a report. Findings are presented as narrative summary.

Study authors (VC and CP) reviewed qualitative data and generated meaning units and codes that were then used to generate, define, and name themes. To ensure reflexivity, the process of analysis was iterative and involved cycles of discussion with members of the wider review team to acknowledge assumptions and gain multiple perspectives on the data. Findings are presented as a narrative synthesis.

## 3 Results

### 3.1 Search Outcomes

The systematic search identified 22,084 references. Once duplications (*n* = 5680) were removed, 16,404 references remained to be screened. After title/abstract screening, 120 references underwent full text review. As the eligibility criteria only permitted research papers for inclusion, any publication that explicitly stated their research ethics committee did not consider the activity research was excluded. This gave a final sample size of 32 references from 29 studies. Multiple publications from a single study were merged at the point of data extraction (see Figure 1: PRISMA flowchart).

**FIGURE 1:**
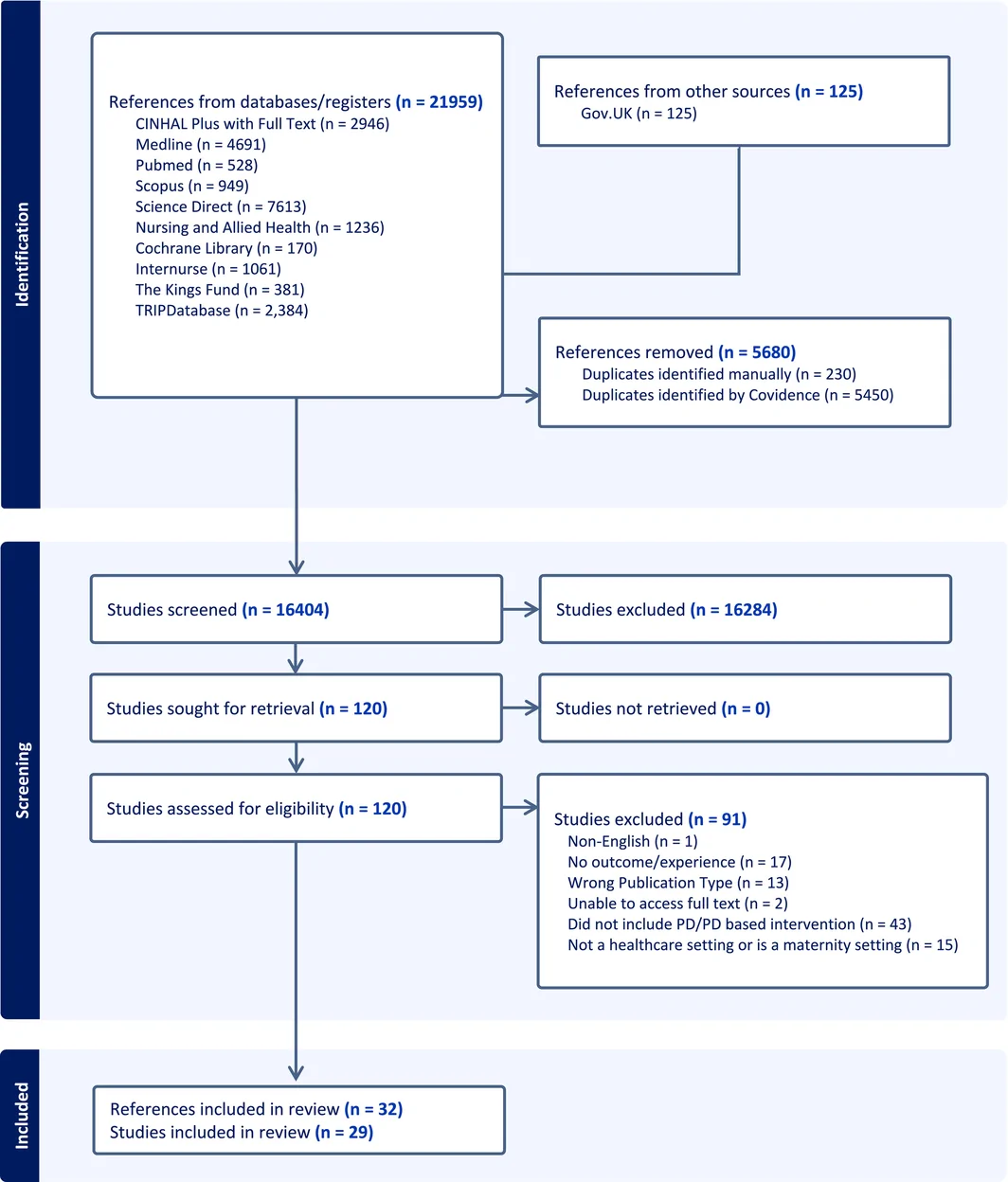
PRISMA flow diagram.

### 3.2 Study Characteristics

The majority of studies presented qualitative methodologies (*n* = 15), followed by mixed methods (*n* = 12) and quantitative (*n* = 2) data. Three of the qualitative studies had two publications each that all met the inclusion criteria of this review. These were merged at the point of data extraction. All but one study was observational in design.

Twelve studies (41.4%) utilised PAR when implementing PD; however, definitive definitions of PD or PAR were not consistently provided. The five (17%) (Garbett and McCormack 2002; Boomer et al. 2019; McCance et al. 2013; Laird et al. 2015; Miskelly and Duncan 2014; Dickson et al. 2018; McCormack et al. 2018; Bagaragaza et al. 2024) studies that did not utilise cyclical processes had not included PAR in their approach. All studies involved nursing staff, either exclusively (*n* = 11) or as part of a wider team (*n* = 18) as participants. Most studies (*n* = 20) were conducted in European countries, with the United Kingdom hosting 10 studies (England = 5; Scotland = 2 and Northern Ireland = 3). Three countries outside of Europe were Australia (*n* = 7), New Zealand (*n* = 1), and Iran (*n* = 1).

Twenty of the 29 studies took place in hospital, followed by nursing homes (*n* = 4), hospice run by a charity (*n* = 3), rehabilitation unit (*n* = 1) and primary care (*n* = 1). Nursing staff were study participants in all studies and for nine studies, nurses were the only staffing group included (Miskelly and Duncan 2014; Bucknall et al. 2022; Bellman et al. 2003; Dahl et al. 2018; Dempsey 2008; Donegan et al. 2021; Hestevik et al. 2019; Moreno‐Poyato et al. 2019; Peet et al. 2022). Patients (Laird et al. 2015; Donegan et al. 2021; Friesen‐Storms et al. 2015; Drayton et al. 2021; Brown and McCormack 2011; Bogerd et al. 2024; Blanchfield and O'Connor 2022; Armitage and Evans 2005; Afshar et al. 2020) and/or family members (Donegan et al. 2021; Friesen‐Storms et al. 2015; Noergaard et al. 2016; Skene et al. 2019) were included for study activities (i.e., surveys, focus groups, interviews) for eleven studies. Information on facilitator(s) was not always explicitly stated. It would appear that most facilitation was done by members of the research team. Across the 29 studies, the duration of the intervention varied from five months (Hestevik et al. 2019) to 2 years (McCance et al. 2013; Laird et al. 2015; Friesen‐Storms et al. 2015; Drayton et al. 2021; Brown and McCormack 2011; Bogerd et al. 2024; Zaforteza et al. 2015; Jackson et al. 2025).

Overall, there was limited information on the characteristics and sample sizes of the study population in most of the papers. See Table 3 for a summary of all included studies and Tables 4 and 5 for a summary of PD Characteristics.

**TABLE 3:** Summary of all included studies.

Reference(s) for study	Methodology	Country	Setting	Age of patients	Patients and/or family members involved	Used PAR	Meet All MMAT (dele: methodological) criteria
Afshar et al. (2020)	Mixed Methods	Iran	Male surgical (orthopaedic and urology) ward in a general hospital	Not stated	Yes—Patient satisfaction questionnaire	Yes	No
Armitage and Evans (2005)	Mixed Methods	Australia	Subacute aged care unit	“On average, 93% of patients admitted to the unit are aged over 65 years”	Yes—Family members experience (information on data collection methods not provided)	No	No
Bagaragaza et al. (2024)	Qualitative	France	Twenty‐one nursing homes	“…care of older persons…”	No	No	Yes
Bellman et al. (2003)	Qualitative	England	Operating Theatre Recovery area in a NHS Trust	Not stated	No	No	Yes
Blanchfield and O'Connor (2022)	Mixed Methods	Ireland	Outpatient units which provide specialist diabetes, endocrinology and/or nephrology care	“…mean age of 74 years…”	Yes—Discovery interviews with patients	Yes	No
Bogerd et al. (2024)	Mixed Methods (only qualitative data presented)	Netherlands	Thirteen general practices	“The age range of patients spanned from 18 to 99 years, with 67% being aged 65 years or older.”	Yes—Cocreation included semi structured patient interviews	Yes	Yes
Boomer et al. (2019)	Mixed Methods	Northern Ireland	Palliative care in a Health and Social Care Trust and private sector nursing homes	Not stated	Yes—Residents' notes were reviewed	No	No
Brown and McCormack (2011)	Mixed Methods	Northern Ireland	Abdominal surgical unit that consisted of two wards	“…older people following abdominal surgery.”	Yes—Pre and postoperative semi structured interviews	No	No
Bucknall et al. (2022)	Quantitative (cluster RCT)	Australia	Thirty‐six inpatient wards at four acute hospitals	Mean age ranged from 63.7 to 65.6 years	Yes—Data extracted from patient medical charts	No	Yes
Dahl et al. (2018)	Qualitative	Norway	Nursing home	Not stated	No	No	Yes
Dempsey (2008)	Mixed Methods	Australia	Two medical wards in a tertiary hospital	“…where approximately 40% of all patients admitted from the Emergency Department are aged over 70 years of age.”	Yes—Data extracted from patient medical charts	No	No
Dickson et al. (2018) and McCormack et al. (2018)	Mixed Methods	Scotland	Hospice	“…support for up to 20 adults over the age of 16.”	Yes—Patient stories and routinely collected data	No	No
Dickinson et al. (2005) and Dickinson et al. (2008)	Qualitative	England	Twenty‐six‐bed unit providing care for older patients with complex discharge needs in an acute NHS Trust.	Not stated	Yes—Semi structured interviews	No	Yes
Donegan et al. (2021)	Mixed Methods	Ireland	Acute hospital/services	Age over 65 years	Yes—Data extracted from patient medical charts and a joint survey of patients and their families	No	No
Drayton et al. (2021)	Mixed Methods	Australia	Subacute rehabilitation unit	Not stated	Yes—Semi structured interviews and questionnaire	No	No
Friesen‐Storms et al. (2015)	Qualitative	Netherlands	Lung unit of a rural hospital	Not stated	Yes—Interviews with patients and informal caregivers	Yes	Yes
Haraldsdottir et al. (2020)	Qualitative	Scotland	Adult hospice	Not stated	Yes—Interviews with patients	No	Yes
Haraldsdottir et al. (2025)	Qualitative	United Kingdom	Eight hospices	Not stated	No	No	Yes
Hestevik et al. (2019)	Quantitative	Norway	Nursing home	“…older residents…”	Yes—Data extracted from patient medical charts	Yes	Yes
Jackson et al. (2025)	Mixed Methods	United Kingdom	Palliative and end‐of‐life care (from level 2 to Level 8)	Not stated	No	Yes	Yes
Ling (Iris) Li et al. (2010)	Qualitative	Australia	General medical ward in a regional tertiary referral hospital	“The patients cared for on the ward include a significant number of frail older people…”	No	Yes	Yes
McCance et al. (2013) and Laird et al. (2015)	Qualitative	Northern Ireland	Wards from four different hospital sites in a Health and Social Care Trust	“Participants were people aged over 18…”	Yes—Narrative interviews	No	Yes
Miskelly and Duncan (2014)	Mixed Methods	New Zealand	District Health Board	Not stated	No	No	No
Moreno‐Poyato et al. (2019)	Qualitative	Spain	Two psychiatric units in a tertiary hospital	Not stated	No	Yes	Yes
Noergaard et al. (2016)	Qualitative	Denmark	Neonatal Intensive Care Unit	Fathers were aged between 29 and 41 years old	Yes—Interviews, focus groups and workshops with parents	Yes	Yes
Peet et al. (2022)	Qualitative	Australia	Single medical‐surgical ward	Not stated	No	No	Yes
Skene et al. (2019)	Mixed Methods	England	Regional Neonatal Intensive Care Unit	Not stated	Yes—Interviews, focus groups and diaries with parents	Yes	No
Venturato et al. (2020)	Qualitative	Australia	Five residential aged care settings	Not stated	No	Yes	Yes
Zaforteza et al. (2015)	Qualitative	Spain	Intensive Care Unit in a tertiary hospital	Not stated	No	Yes	Yes

**TABLE 4:** Practice Development Characteristics.

	Context of intervention	Evidence‐based practice	Continuous education and training	Quality improvement initiatives	Patient centred care	Technology integration	Leadership support and engagement	Peer support and mentoring	Measurement and evaluation	Flexibility and adaptability	Cultural competence	Reflection in practice
Afshar et al. (2020)	Yes	Yes	Yes	Yes	No	No	Yes	No	Yes	Yes	No	Yes
Armitage and Evans (2005)	Yes	Yes	Yes	Yes	Yes	No	No	Yes	Unclear	No	No	Unclear
Bagaragaza et al. (2024)	Yes	Yes	Yes	Unclear	Yes	No	Yes	Yes	Yes	Yes	Yes	Yes
Bellman et al. (2003)	Yes	Yes	Yes	Yes	Yes	No	Yes	Yes	Yes	Yes	No	Yes
Blanchfield and O'Connor (2022)	Yes	Yes	Yes	Yes	Yes	No	No	Yes	Yes	No	No	Yes
	Yes	Yes	Yes	Yes	Yes	Yes	Yes	Unclear	Yes	Yes	No	Yes
Boomer et al. (2019)	Yes	Yes	Yes	No	Yes	No	Yes	Unclear	Yes	Yes	No	No
Brown and McCormack (2011)	Yes	Yes	No	No	Yes	No	Yes	Yes	No	Yes	No	Yes
Bucknall et al. (2022)	Yes	Yes	Yes	Unclear	Yes	Yes	Yes	Yes	Yes	Unclear	No	No
Dahl et al. (2018)	Yes	Yes	Unclear	Yes	Yes	No	Yes	No	Yes	Unclear	Yes	Yes
Dempsey (2008)	Yes	Yes	Yes	Yes	Yes	No	Yes	No	Yes	Yes	No	No
Dickson et al. (2018) and McCormack et al. (2018)	Yes	Yes	Yes	Yes	Yes	No	No	Yes	Yes	No	No	Yes
Dickinson et al. (2005) and Dickinson et al. (2008)	Yes	Yes	Yes	Yes	Yes	No	No	Yes	Yes	Yes	Yes	No
Donegan et al. (2021)	Yes	Yes	Yes	Yes	Yes	No	Yes	Unclear	Yes	Yes	No	No
Drayton et al. (2021)	Yes	Yes	Yes	Yes	Yes	No	Yes	Yes	Yes	No	Yes	Yes
Friesen‐Storms et al. (2015)	Yes	Yes	No	No	Yes	Unclear	Yes	No	Yes	Yes	No	Yes
Haraldsdottir et al. (2020)	Yes	Yes	Yes	No	Yes	No	Yes	No	Yes	Yes	No	Yes
Haraldsdottir et al. (2025)	Yes	No	No	Unclear	Yes	No	Yes	Yes	Yes	Yes	Unclear	Yes
Hestevik et al. (2019)	Yes	Yes	Yes	Yes	Yes	No	No	Yes	Yes	No	No	Yes
Jackson et al. (2025)	Yes	Yes	Unclear	Unclear	Yes	No	Yes	Yes	Yes	Yes	Yes	Yes
Ling (Iris) Li et al. (2010)	Yes	Yes	No	No	Yes	No	Yes	Yes	Yes	Yes	Unclear	Yes
McCance et al. (2013) and Laird et al. (2015)	Yes	Yes	Yes	Yes	Yes	No	No	Yes	Yes	No	No	Yes
Miskelly and Duncan (2014)	Yes	Yes	Yes	No	Yes	Yes	Yes	Yes	Yes	No	Yes	Yes
Moreno‐Poyato et al. (2019)	Yes	Yes	Yes	Yes	Yes	No	No	Yes	Yes	Yes	No	Yes
Noergaard et al. (2016)	Yes	Yes	Yes	Unclear	Yes	No	No	No	Yes	Yes	No	Yes
Peet et al. (2022)	Yes	Yes	Yes	Yes	Yes	No	No	No	Yes	Yes	No	Yes
Skene et al. (2019)	Yes	Yes	Yes	Yes	Yes	No	No	No	Yes	Yes	Yes	Yes
Venturato et al. (2020)	Yes	Yes	Yes	Yes	No	No	Unclear	Unclear	Yes	Yes	Unclear	Yes
Zaforteza et al. (2015)	Yes	Yes	No	No	Yes	No	Yes	Yes	Yes	No	No	Yes

**TABLE 5:** Practice Development Interventions.^a^

	Practice Development Interventions			
Afshar et al. (2020)	Problem Identification “There were 20 interviews with staff nurses, head nurse, supervisors, matron and surgeons. Four patients were also interviewed… All the nurses completed the Conditions of Work Effectiveness Questionnaire‐II and job satisfaction questionnaires.” 2 Planning and Action “In four focus groups, the problems and possible changes were discussed with nurses. The suggestions were analysed, and the feasibility of the plans was reviewed. Three change plans that were more applicable were selected by consensus in action research team. The action plan was designed for each change…. The progress and possible modifications were evaluated in three focus groups…” 3 Reflection and Learning “During action research and in the end of the programme, the reflections were gathered through focus groups and interviews… all the nurses completed the questionnaires once again… patients also completed the patient satisfaction questionnaire.”			
Armitage and Evans (2005)	Problem Identification “…the nursing team kept a record of adverse incidents for patients with dementia who were dying over a six‐month period… Examining current practice it this way it because clear that a number of factors contributed to a poor outcome… An education plan was developed and implemented by all members of the nursing team…nurses than met with local GPs…” 2 Implementing Strategies “The education sessions were repeated over a period of 3 months… These meetings resulted in a range of practice changes… The area palliative care service was contacted to discuss its role in the care of patients… An offer to make available the services…was welcomed… The team social worker conducted education sessions…” 3 Outcomes and Evaluation “Evaluation of the changes to practice were undertaken both progressively and 3 months after the implementation…”			
Bagaragaza et al. (2024)	“The Padi‐Palli critical companionship model was built on three fundamental pillars: (1) sharing knowledge and experiential clinical experience, co‐constructing new knowledge, facilitating reciprocity in learning and transferring acquired learning immediately into practice; (2) valuing what already exists such as practices, workplace culture, resources and the organisation, and caring for other professionals; and (3) encouraging new initiatives, challenging the status quo, reflecting on one's practice, participating in role modelling and providing emotional and cognitive support. The humanistic educative values of respect, openness, creativity, emphatic understanding and person‐centredness not only act as a resource and facilitate learning that responds to learner needs26 but also form the key principles that guide the critical companions as they provide training and support. To better align with the particular work environment of the NH with its diverse professional groups, we added an interprofessional dimension to Padi‐Palli critical companionship to enable interprofessional dialogue as it is key to palliative care practices. Hence, unlike the original critical companionship framework that recommends a dual relationship, the Padi‐Palli critical companionship model privileged the interplay between dual and multidisciplinary facilitative relationships. The learning environment emphasised experiential learning at the bedside of residents and within care units instead of formal classroom‐based education.”			
Bellman et al. (2003)	Initiating the study “The study evolved…to advance the standard of care in the Recovery Suite by updating her staff and concurrently involving them in shared learning and practice development… Clinical, educational and research support for the project was addressed through a tripartite arrangement… They explored the basis of their collaboration, i.e., their beliefs and values regarding practice development – for individual nurses, the clinical team, the organisation, the involvement of users. They also explored the nature of support for one another and the co‐researchers, and particularly the need for ongoing support from senior managers in all three organisations.” 2 Data collection and analysis “To systematically explore the objectives of the study and capture the process and outcomes, five approaches to data collection were undertaken: audio‐taping of the co‐researchers' action learning sets; steering group meetings; project board meetings; other stakeholder meetings field note recordings research journals audio‐taped group interviews e‐mails The multi‐method approach provided an opportunity to use different sources of evidence in an effort to develop converging lines of inquiry, sometimes termed triangulation….” 3 The evolving process “The ongoing involvement of key stakeholders was an essential part of the action research process. The open communication process both enabled and empowered the front‐line co‐researchers to directly influence the development of the study… An enabling strategy, undertaken at the first action learning set, was an individual and team values clarification exercise…” 4 Process outcomes “The co‐researchers' collaborative learning and working resulted in the initiation of three patient‐focused projects: …Analysis of the data enabled the explication of 27 process outcome categories. The 27 categories have been grouped into 5 themes which reflect change and development for the co‐researchers”			
Blanchfield and O'Connor (2022)	“…a series of three cycles of enquiry [reflect, plan, act, observe]… six discovery interviews [patients] and four focus groups [staff]…The collection and analysis of discovery and focus group interviews yielded 14 major themes which were used by the PAR group to inform discussion and action for the second cycle of enquiry… Three PAR group meetings were convened… A key component of the PAR cycle is a reflection in which all participants are co‐researchers and equally represented… Using group consensus the following eight actions were formulated in cycles 1–3 which led to the establishment of the new combined care… The final cycle of enquiry was undertaken…with a primary focus to identify clinical outcomes and the contribution of advanced practice nursing to combined type 2 diabetes and CKD care…”			
Bogerd et al. (2024)	“…consisting of four plan‐act‐observe‐reflect‐adjust‐cycles… Participating actors were GPs, PNs, patients from the target group, the affiliated care cooperation, representatives of a health insurer and researchers. A co‐creation team was formed and consisted… the researchers acted both as participants and facilitators… an initial exploratory phase established the starting point, followed by four iterative plan‐act‐observe‐reflect‐adjust cycles lasting approximately 6 months each. Each cycle comprised a 4‐month action phase and a 2‐month ‘evaluation and adjust phase’… The subsequent ‘evaluation phase’ involved reflection on the actions taken. Data collection and analysis involved extracting pseudonymised routine care data from the EMR, conducting focus groups with healthcare providers…and patient interviews…Work satisfaction…was measured pre and at the end of the final co‐creation cycle.”			
Boomer et al. (2019)	“The practice development methods used included: working collaboratively to agree roles and responsibilities and the focus for staff and practice development; being person‐centred and exploring this in the practice context; clarifying values, focus and vision; working collaboratively; and developing relationships to encourage participation in the homes… These methods were intended to promote ownership of the developments in the nursing home setting, thereby creating the drive to sustain and promote a culture where palliative and end‐of‐life care for residents would continue to develop after the end of the programme… The education element of the programme was delivered… Participants were asked to undertake practice development activities in their nursing homes in between the sessions… Programme activities gave the project team…an idea of the participants' level of knowledge and skill, education needs and insight into the culture of care, enabling them to adapt the content accordingly. The education sessions used a mixture of practice development tools and approaches, traditional slide presentations, and group discussion and reflection to encourage a person‐centred approach to planning and providing care.”			
Brown and McCormack (2011)	“Adopting the principles of co‐operative inquiry…all consenting nursing staff had the opportunity to work in focus groups, facilitated reflective sessions, ad hoc reflective sessions, and consolidation workshops to explore their experiences and reflect together… Completing two episodes of non‐participant observation of nursing practice midway and at the end of the project… At the end of each observation period, data were shared with the nursing team and reflective discussions were recorded… Inviting six older patients to participate in pre and postoperative semi‐structured interviews… Completing the NWI‐R Questionnaire by…nursing staff to provide further insight into the culture and nurse decision making in the unit… During focus groups, the ethnographic study findings were discussed with participants in order to establish their credibility… nursing staff initiated a whole‐team workshop with the aim of consolidating data gathered; developing shared values and beliefs; developing a shared language; and identifying action cycles and practical strategies for change… To work on these action cycles, nursing staff chose to form small reflective groups that were entitled reflective sessions…”			
Bucknall et al. (2022)	“…receive standard clinical practice guideline (CPG) dissemination…or facilitated implementation for 6 months following standard dissemination… Core [facilitated implementation] components: – Training: Being a facilitator, processes of facilitation, toolkit of techniques, knowledge of the patient problem and national guideline requirements. Review of hospital policy, VS triggers for escalation and nursing interventions. – Identification of ward barriers and enablers: individual, discipline, ward and organisation. – Monthly ward audits with feedback of results to nurse managers and staff via posters, presentations and discussions of areas for improvement. Monthly HFLIP facilitation support by external expert facilitator reviewing audit results, how to respond to ward issues, questions and concerns. – Toolkit of facilitation techniques…provided to HFLIPs: Clarify and engage; assess and measure; action and implementation; review and share. – Techniques included: interactive education, case presentations, individual discussions, reminder posters, working with clinicians during assessments, interdisciplinary discussions and ward audits with feedback. Each facilitator used the same techniques. Adaptable [facilitated implementation] components: Facilitation techniques were selected from the toolkit and tailored depending on the context such as patient management problems, ward audit results, ward staff needs and their availability”			
Dahl et al. (2018)		**Educational intervention**	**Research methods used during the formal intervention sessions**	**Research methods used in the workplace and in between the formal sessions**
	Step 1	Establishing contact with the nursing home leaders and clarifying the premise for the practice development		
	Step 2	Two‐day seminar: ‘Trust before restraint’	Participant observation and reflective journal	Participant observation, informal interviews
	Steps 3, 4 and 5	Three sessions of facilitation	Participant observation and reflective journal	Participant observation, formal and informal interviews
	Step 6	Seminar on communication skills (Marte Meo method) in addition to the facilitation	Participant observation and reflective journal	Participant observation, formal and informal interviews
	Step 7	Facilitation	Participant observation and reflective journal	Participant observation, formal and informal interviews
	Closure	A closure of the study by evaluating and discussing the process, as well as arranging a seminar to discuss outcomes related to workplace learning		
Dempsey (2008)	“The pilot project was conducted in rounds or phases, each building on the results as may be seen in the following description: Rounds: Evaluation of current measurement of risk instrument (chart); Clinical comparison of new and current instruments; Trial use of new instruments; Test for inter‐rater reliability, sensitivity, specificity and predictive values; Clinician satisfaction using a visual analogue scale.”			
Dickson et al. (2018) and McCormack et al. (2018)	“Phase 1: Looking The ‘first stage in a quality action research study is to establish the basic values underpinning the care in a given area’…. During this phase we explored the realities and context of mealtime care by: Observing mealtimes. This enabled us to see some of the issues which were having an impact on patient care at mealtimes. Collecting the perspectives of staff, patients and other visitors to the ward about mealtimes. Focus groups were held with staff, interviews with patients and a comments box was placed on the ward for patients, staff and visitors to record comments and ideas. Think and act: Phase 2 This phase incorporated the ‘thinking and action’ phase of the project. We used action learning groups with staff, as well as role modelling of good practice and encouragement of ‘reflection‐on‐action’… through ‘facilitation of learning’ Phase 3: Evaluation This phase involved evaluation of the project, by repeating the data collection of Phase 1”			
Dickinson et al. (2005) and Dickinson et al. (2008)	*Development programme for practice development facilitators* “The programme was designed to reflect Garbett and McCormack's (2002) practice development model. The principles of practice development include values clarification and development of a shared vision as the starting point, while the adoption of systematic and rigorous evaluation of process and outcomes has the focus of developing person‐centred cultures. Facilitator development is central. Four workshops were facilitated over a period of several months, focusing on: Exploring person‐centredness and enhancing knowledge and understanding of practice development Developing a shared vision Evaluating effective workplace cultures Examining process and outcome methods of evaluation Facilitating active learning Celebrating success” *Programme of practice development to further the development of a culture of person‐centred practice* “The programme was structured over 12 months and centred around 10 ‘programme days’ of learning and development. Each of these days incorporated three hours of facilitated active learning with the core group on the identified themes. While the programme appears linear in design, each session incorporated elements of previous learning and responded to the learning and development needs of the participants.”			
Donegan et al. (2021)	“We used a combination of person‐centred and Lean Six Sigma methodologies. The principles of person‐centredness, adopted throughout the project, ensured that the entire team recognised the following aspects of person‐centred planning of care: It starts with the person's perspective on his or her life It entails a creative approach that asks what is possible, rather than assuming common understandings and limiting itself to what is available It takes into consideration all the resources available to the person… The project included seven stages: Reviewing current processes nationally Understanding reasons for admission to hospital Examining lengths of stay Determining patients' discharge destinations Process mapping Chart review Patient and staff feedback”			
Drayton et al. (2021)	“The six phases are: Preparation—engaging teams Assessment—gathering information about care and culture Feedback—critically reflecting and identifying themes Planning—prioritising and actioning themes Action, ongoing—implementing and evaluating actions Re‐assessing, every 2 years—re‐gathering information about care and culture at the end of each cycle”			
Friesen‐Storms et al. (2015)	“The method used in this study was a participatory action research design with cyclical activities involving observing, reflecting, planning and acting. The observing and reflecting phases provided information for subsequent planning… To critically review the existing situation, we started the observing phase with a working group consisting of the researcher (AM), four nurses of the lung unit, the unit's manager and a staff member of the hospital's educational department. We reflected on the current practice situation and the barriers and facilitators for implementing EBP, to select strategies for implementing EBP addressing barriers and facilitators, and the particular contextual situation regarding EBP. The nurses intended to use the development and implementation of a unit‐specific EBP protocol for nursing discharge as a vehicle to implement EBP. We continuously evaluated the process in the working group by means of observing and reflecting, and made a summative evaluation at the end of the project.”			
Haraldsdottir et al. (2020)	“…practice development methodology was applied with the key aim of transforming the context and culture within the hospice. This methodology includes processes such as: developing shared values amongst team members; having a shared vision for ideal practice; developing team relationships; using work based reflective learning strategies; engagement in critical questioning; and adopting a systematic approach to changing everyday practice. The key aim is to direct attention to the micro‐level of practice, where healthcare is experienced and provided. The principles of participation, collaboration and inclusivity always underpin evaluation activities in practice development. The focus is on engaging practitioners, through creative learning methods and skilled facilitation, in critical questioning about their own practice and enable them to answer their own questions.”			
Haraldsdottir et al. (2025)	“The programme comprised 7 workshop days delivered over a 7‐month period. Of these, two workshops were held face‐to‐face‐one at the beginning and one at the end of the project. Each hospice received mentorship from one of three facilitators throughout the programme… The programme was designed to be flexible, allowing each participant to develop a contextualised practice development plan tailored to their own hospice setting…the focus was placed on the culture and context of care within each participant's practice area, with the responsibility for action falling to the participants themselves. The aim was to support the development of the participants' personhood, values, and beliefs as the foundation for their practice development and learning, ultimately enabling them to become person‐centred facilitators in their own settings.”			
Hestevik et al. (2019)	“The PAR project was a multidisciplinary collaboration led by representatives from two university colleges and a nursing home ward. The project aimed to improve the participating healthcare providers' competence on assessing nutritional status, providing quality nutritional care and documenting nutritional information in the medical records. Seven project meetings (lasting 1.5–2 h) were arranged regularly over a 5‐month period and were led by one teacher from each of the university colleges, one nutritionist and one nurse. The project meetings were dialogue based and alternated between practical experience and theoretical reflection. All participants in the project were co‐researchers. The participants were able to acquire personal experience, to reflect upon this experience together with the other participants and to receive feedback and gain different perspectives within the multi‐professional team. The participants identified professional, organisational and ethical challenges to the nutritional care practice in the nursing home. These challenges were reflected upon and possible solutions on how to improve the practice were agreed on. In the next sessions, experiences from implementing this in practice were discussed and adjustments were made.”			
Jackson et al. (2025)	“Central to collaborative ways of working together with…practitioners at all levels of the organisation were the core principles of practice development critical to authentic co‐design. Well‐constructed co‐designed projects facilitate collaboration and a team approach to the design, evaluation and application of solutions which transform practice and are more likely to be sustained.”			
Ling (Iris) Li et al. (2010)	“The focus of the practice development programme was to enable nursing teams to explore the concept of person‐centredness within their own clinical setting, in order to improve care delivery. The programme was delivered over 2 years and the structure comprised facilitated activities in line with a practice development approach… The programme was delivered through a series of facilitated workshops (*n* = 5), with ongoing monthly support provided through a project team. Each workshop focused on key themes including: Promoting an understanding of person‐centredness Developing a shared vision Determining the quality of the user experience Systematically developing practice Celebrating success “The Person‐Centred Practice Framework …has guided and structured studies that have focused on the development, implementation and evaluation of clinical practice in a variety of contexts and care settings… The Framework…underpinned a commissioned systematic practice development programme.”			
McCance et al. (2013) and Laird et al. (2015)	“First Research Meeting: Establishment of group process… During the process, we acknowledged the need to make decisions for improvement and act upon these in our daily practice and care… We had 13 weekly PAR group meetings over 6 months… The agenda for each the meeting was set by the PAR group so that the agreed upon action evolved over time from our meetings… In the ‘looking’ phase, the academic group members lead the group, with one acting as a group facilitator. They invited us to share an experience we had with caring for someone with delirium. Guided by the facilitator, we then reflected on all of the stories about our experiences and the evidence based literature. Using the stories and the literature we reflected on the issues and explored possibilities for action that could be implemented on our ward. The story telling approach was an excellent approach that encouraged us to interact with each other by sharing ideas and stimulating new insights into the topic… Based on our group discussions and reflection of our practice stories in the ‘thinking’ phase the academics introduced us to Inouye's Hospital Elder Life Program which focuses on delirium prevention… Often the ‘thinking’ part of the meetings involved us being able to express our thoughts and concerns… Action – the outcome of the research…”			
Miskelly and Duncan (2014)	“The main principles underlying PD…are… Practice development is values based and an important aspect of Pebbles is related to participants articulating their own philosophies and values; for example, why they chose nursing and midwifery as a profession, and the values they relied on in order to do their job. Part of this discovery phase was referred to as ‘the heart of their practice’. Facilitation underpins PD and is used to dispel and challenge the taken‐for‐granted attitudes people develop towards their individual practice and work environments, encouraging instead reflection, critical analysis, discussion and debate, as well as an ‘enabling’ culture… Pebbles was run over a 6‐month period… The content of the programme has been continually revised since its inception…”			
Moreno‐Poyato et al. (2019)	“…four focus groups were held, lasting from 1.5 to 2 h. In addition, 19 reflective diaries were collected throughout the process. To monitor the research process both descriptively and methodologically and to help integrate theory and practice, we also used the investigators' field diaries… The study procedure was conducted in two phases…. The first phase consisted of an initial stage (stage 0) in which we analysed the situation and the study context. In the next four stages, the participants designed guidelines to self‐observe their practice, and then conducted and registered their self‐observations. Next, they described the contrast between their observed practice and the scientific evidence. In the second phase, the nurses proposed strategies in relation to the evidence, and then implemented and evaluated them. Finally, they narrated aspects related to the PAR process…”			
Noergaard et al. (2016)	“The exact type and number of activities were not decided a priori; rather, they were decided during the process in order to engage relevant stakeholders in an appropriate and meaningful way… The actions and reflections were guided by the wish to generate (i) knowledge of the everyday lives of and the attitude towards fathers in the unit; (ii) detailed knowledge of the fathers' experiences, needs, and wishes; (iii) new ideas and inspiration; and (iv) actions towards a father‐friendly NICU. Initially, participant observation provided preliminary knowledge of the fathers' needs and the NICUs' culture. The participant observation implies proximity to the stakeholders and the starting point to ground the PAR approach in the perspective and interests of those immediately concerned. The purpose of the participant observation was to obtain knowledge of (i) the fathers' and mothers' presence and actions in the everyday life in the NICU and (ii) the staff's daily activities, how they supported the fathers, and how they cared for children and their families.”			
Peet et al. (2022)	“This research undertook a process of engagement and contextual exploration, transformative action cycles and immersive reflexive evaluation… Considering the co‐learning approach of ePD research, we gave careful attention to our positionality as insider (as nurses) outsider (external researcher) roles… This was fully appreciated during an initial 12‐month period of researcher immersion… A swirling process of action cycles supported practice change by working with people rather than on people. This commenced with ePD workshops where frontline nursing staff were recognised as experts in their own context and placed at the centre of their patient safety enquiry. The formation of an Action Learning Set (ALS) continued the transformative journey…”			
Skene et al. (2019)	“The study comprised three phases. In Phase 1, an understanding of the context of parental involvement in care was established, factors influencing parent‐infant relationships were identified and evidence‐based interventions developed to be implemented across the unit. In Phase 2, three action research cycles allowed the interventions to be tested and further refined through a process of planning, acting, observing, reflecting and re‐planning. Phase 3 involved appraising the success of the interventions.”			
Venturato et al. (2020)	“The TOrCCh intervention is thus a systematic change process, facilitated in this pilot study by research staff working with facility work teams who acted as “champions” within each site. The intervention is based upon a participative change cycle and includes the formation of work teams, development of knowledge and associated skills regarding culture change, identification of an area of practice for change, implementation of the change, evaluation and reflection. This methodology is informed by an action research/action learning approach that positions learning and reflection at its centre and that is focused on both processes and outcomes… The action research method used in the TOrCCh intervention is based on a “QPAR” (Question, Plan, Act and Reflect) cycle…”			
Zaforteza et al. (2015)	“…had five phases based on the reflection–action cycles… Diagnosis of care provided to patients' families, characterised by individual and collective reflection processes. A consensus document described the care situation and the unit's highly adverse attitude towards changing it. Selection of change proposals, characterised by collective reflection. Consensus was reached on four change initiatives related to caring for family members of critically ill patients. Design of proposed changes, characterised by dialogic processes. All 60 participants worked on teams, with each group managing one of the four initiatives. Implementation of change proposals, characterised by dialogue and action. Each team sought alliances and strategies to support their initiative, identifying individuals with decision‐making authority. Evaluation of the PAR process, characterised by individual and collective reflection. Evaluation was conducted at 3 months and 1 year of follow‐up.”			

^a^ Text provided are quotations from each cited paper.

### 3.3 Study Risk of Bias

Eighteen studies (McCance et al. 2013; Laird et al. 2015; Bucknall et al. 2022; Bellman et al. 2003; Dahl et al. 2018; Hestevik et al. 2019; Moreno‐Poyato et al. 2019; Peet et al. 2022; Friesen‐Storms et al. 2015; Bogerd et al. 2024; Noergaard et al. 2016; Zaforteza et al. 2015; Dickinson et al. 2005; Dickinson et al. 2008; Haraldsdottir et al. 2020; Ling (Iris) Li et al. 2010; Venturato et al. 2020) met all methodological quality criteria for their respective study design. Although there were thirteen mixed methods studies, two (Bogerd et al. 2024; Jackson et al. 2025) presented qualitative data only and were assessed as a multi‐methods qualitative study. Nine mixed methods studies provided limited information on quantitative methods to enable a full assessment (Boomer et al. 2019; Miskelly and Duncan 2014; Dickson et al. 2018; McCormack et al. 2018; Drayton et al. 2021; Brown and McCormack 2011; Blanchfield and O'Connor 2022; Armitage and Evans 2005; Afshar et al. 2020; Skene et al. 2019). As a result, we were unable to determine if the quality criteria of quantitative methods were adhered to and therefore could not assess for integration, divergences and inconsistencies between qualitative and quantitative components and/or results. Two mixed methods studies (Dempsey 2008; Donegan et al. 2021) that did meet the quantitative methodological quality criteria did not adequately address divergences and inconsistencies between quantitative and qualitative results or demonstrate that the integration of qualitative and quantitative components was adequately interpreted. All mixed methods studies met the qualitative methodological criteria assessments. MMAT assessment was used to inform interpretation and methodological quality; no studies were excluded based on quality assessment alone.

### 3.4 Qualitative Findings

Reflexive analysis of qualitative data generated three broad themes with 13 corresponding subthemes. (1) Shaping Practice: the interplay of organisational context, culture, and structure. (2) Enablers and constraints in Practice Development. (3) Practice Development for collective empowerment (see Figure 2).

**FIGURE 2:**
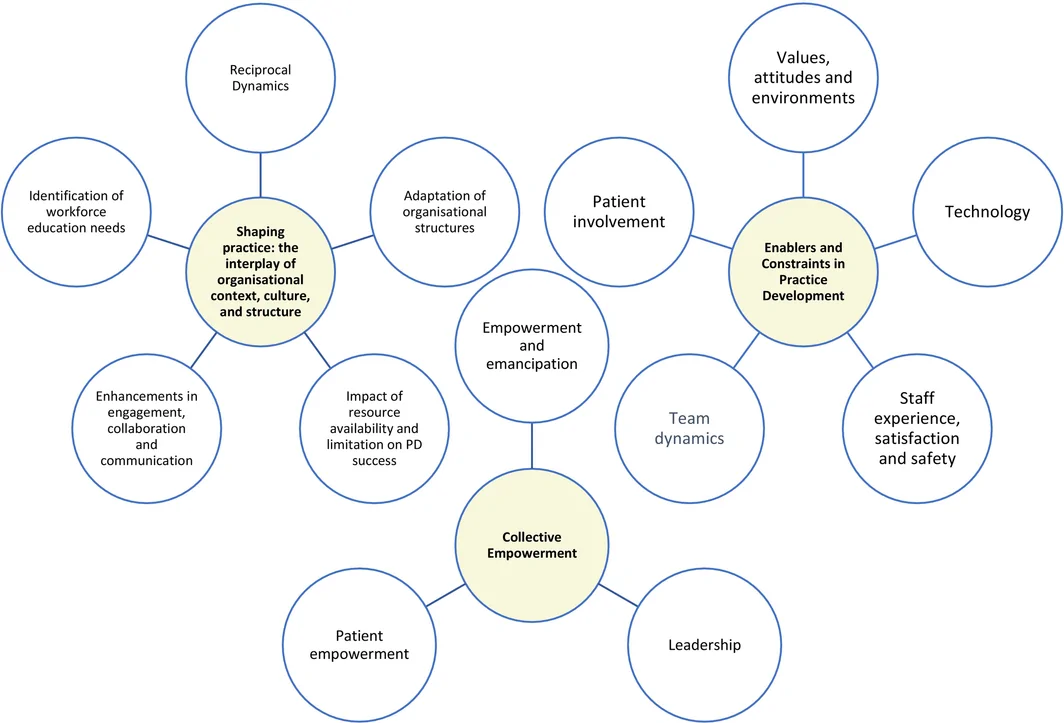
Qualitative theme map.

#### 3.4.1 Theme 1: Shaping Practice: The Interplay of Organisational Context, Culture, and Structure

##### 3.4.1.1 Reciprocal Dynamics

A key principle of PD is that it is enabled by methods and processes contextualised to the work environment (Bradd et al. 2017). Contextual sensitivity was present in all 29 studies included in this review. Attention to organisational structures and workplace culture is noted to be a facet of this contextual sensitivity, with PD activities facilitating the exploration and recognition of existing workplace cultures, prior to the PD intervention (Bagaragaza et al. 2024; Bellman et al. 2003; Friesen‐Storms et al. 2015; Brown and McCormack 2011; Blanchfield and O'Connor 2022; Afshar et al. 2020; Jackson et al. 2025; Haraldsdottir et al. 2020). These activities enabled recognition of existing perspectives and attitudes to care amongst staff (Blanchfield and O'Connor 2022), the impact of external influences on services (Bagaragaza et al. 2024; Blanchfield and O'Connor 2022) the impact of environmental constructs (Haraldsdottir et al. 2020; Haraldsdottir et al. 2025) and psychological safety (Brown and McCormack 2011) as key workplace culture features identified by PD activities.

Evidence of successful workplace culture change as a result of PD is demonstrated in projects that use PD alone (Bagaragaza et al. 2024; Armitage and Evans 2005; Noergaard et al. 2016; Haraldsdottir et al. 2025) and together with action research approaches such as PAR (Bellman et al. 2003; Moreno‐Poyato et al. 2019; Brown and McCormack 2011; Skene et al. 2019; Jackson et al. 2025). Enhanced opportunities for collaboration and communication may support successful workplace culture change (Bagaragaza et al. 2024; Bellman et al. 2003; Moreno‐Poyato et al. 2019; Peet et al. 2022). Culture change may also be underpinned by responsive practice changes that occur as a result of PD, such as enhancing inclusion in handover processes (Boomer et al. 2019), and including multiple perspectives in the development and implementation of new protocols (Friesen‐Storms et al. 2015). PD appears to enhance engagement amongst nursing teams, which may increase motivation for nurse‐led practice change and action for quality improvement (Bellman et al. 2003; Brown and McCormack 2011; Armitage and Evans 2005; Skene et al. 2019). The engagement of nursing staff in examining practice contexts may lead to the development of shared and lasting visions which underpin successful workplace culture change.

There is evidence that PD activities may create an environment which fosters new perspectives and approaches (Skene et al. 2019; Haraldsdottir et al. 2025) which in turn support the creation of more open, reflective and action‐oriented cultures. PD may offer opportunities to enhance transparent, honest environments that are free of judgement (Dickson et al. 2018; McCormack et al. 2018). Additionally, the inclusion of different individuals and perspectives, such as service users, in PD may further develop more open, reflective and action‐oriented cultures (Noergaard et al. 2016). Where PD is combined with other approaches or philosophies, such as PAR and emancipatory action research, culture change appears particularly tangible. When PD and PAR were combined, results were perceived as “quite stunning […] It has given the whole place a sparkle, an energy injection, an enthusiasm (Venturato et al. 2020), whilst emancipatory PD was perceived to enable the pro‐found impact of small‐scale, microsystem level practice transformation” (Peet et al. 2022). A small number of papers suggested barriers to PD achieving successful culture change. Some highlighted the reticence of some staff to relinquish existing power structures and hierarchies as a barrier to PD, owing to individual's fear (Bellman et al. 2003) or historical attitudes towards medical power (McCance et al. 2013; Laird et al. 2015). Whilst newer staff in medicine and nursing may recognise the need for culture change (McCance et al. 2013; Laird et al. 2015), PD may not always achieve this within the project time frame (Friesen‐Storms et al. 2015).

A key feature of culture noted in the included papers was a focus on person centredness. This feature was noted both as an explicit aim of PD (McCance et al. 2013; Laird et al. 2015; Dickson et al. 2018; McCormack et al. 2018; Dahl et al. 2018; Donegan et al. 2021; Drayton et al. 2021; Haraldsdottir et al. 2020; Haraldsdottir et al. 2025), and also in more nuanced ways, such as staff identifying ways to move from a condition‐focused to a person‐centred approach (Dahl et al. 2018; Blanchfield and O'Connor 2022; Jackson et al. 2025) and enhancing family‐centredness in neonatal intensive care units (Noergaard et al. 2016; Skene et al. 2019). Where a focus on enhanced person‐centredness was embedded as a shared vision amongst staff, this also enhanced staff's perceptions of themselves and each‐other as staff realised person‐centeredness as a value “which places the individual at the heart of everything we do” (Dickson et al. 2018; McCormack et al. 2018).

##### 3.4.1.2 Adaptation of Organisational Structures

Organisational structures are noted as a facet of organisational culture. PD facilitates multiple perspectives in an action‐oriented approach to problem solving and practice enhancement and thus may enable successful user‐led re‐design of care environments which, in turn, have dynamic ripple effects upon workplace cultures and wider contexts. Structural changes made as a result of PD included the permanent redesignation of existing facilities and implementation of new care processes to better meet service requirements (Donegan et al. 2021), and user‐led re‐design of work processes and tasks such as re‐design of medical reviews (Blanchfield and O'Connor 2022); drug rounds and mealtimes (Dempsey 2008); handovers (Boomer et al. 2019; Brown and McCormack 2011; Venturato et al. 2020); and nursing functions (Moreno‐Poyato et al. 2019). A component of these structural changes appears to be increased opportunities for multidisciplinary communication and collaboration in patient care, which reduces additional workload, increases efficiency and enhances care experiences (Bagaragaza et al. 2024; Donegan et al. 2021; Friesen‐Storms et al. 2015; Haraldsdottir et al. 2025). Being action‐led and driven by participatory methods, the changes to services and structures driven by PD may depart from more traditional approaches, which historically take a “top‐down” approach (Moreno‐Poyato et al. 2019; Blanchfield and O'Connor 2022). Conversely, in PD, organisational and structural change is often driven from the “bottom up”; a premise which challenges historical power structures and is recognised as “frightening for some*”* (Bellman et al. 2003).

##### 3.4.1.3 The Impact of Resource Availability and Limitation on PD Success

The impact of resource availability and limitations on the implementation and success of PD is notable in the included papers. In the challenging context of a pressured healthcare service, resource limitations were also noted to influence the impact and effectiveness of PD initiatives. This included perceptions of lack of time for PD (McCance et al. 2013; Laird et al. 2015; Friesen‐Storms et al. 2015); requirement for increased effort and energy (McCance et al. 2013; Laird et al. 2015); staffing pressures (McCance et al. 2013; Laird et al. 2015; Dickson et al. 2018; McCormack et al. 2018), conflicting priorities and the challenges of an evolving context (McCance et al. 2013; Laird et al. 2015) were felt to limit the potential impact of PD initiatives. In some cases, the PD process itself identified resource limitations such as staffing shortages, prior to PD initiatives being implemented (Afshar et al. 2020). These resource limitations and external pressures may limit the ability of staff to engage in PD:

Because of staff shortages…. I know for a fact the staff haven't got near the information files to read them because they just don't have the time, it's a way down the list and trying to put our action plan into place is really difficult, we are wanting to move forward with […] but we don't have the time. (McCance et al. 2013)

Some papers highlight how PAR enabled identification of mitigations for resource limitations, such as involving nursing students in patient education (Afshar et al. 2020) and succession planning (Blanchfield and O'Connor 2022), however it is unclear how these measures created sustained impact in the longer term (beyond the PD project timeline).

Practical resources were also noted as an output of PD. As PD engages those who experience the “sharp end” of care in the development of practice, this may result in the development of practical care resources that more closely meet the needs of those they are intended for (Dempsey 2008; Donegan et al. 2021). In two studies, PD facilitated the need for and creation of additional practical resources to support communication with patients and families, which support timely communication and information sharing (Donegan et al. 2021; Venturato et al. 2020).

##### 3.4.1.4 Enhancements in Engagement, Collaboration and Communication

The importance of consistent engagement of staff throughout the PD journey is evident in the included papers. Some barriers to staff engagement were identified, including perceptions that PD had not changed individual practice (Dickson et al. 2018; McCormack et al. 2018), difficulty maintaining consistency of the PD group amid rostering challenges (Venturato et al. 2020), and difficulty translating theoretical approaches into practice (Friesen‐Storms et al. 2015). Allocated time and opportunity for reflection is identified as a key PD tool (McCance et al. 2013; Laird et al. 2015; Bellman et al. 2003; Dahl et al. 2018; Moreno‐Poyato et al. 2019; Peet et al. 2022; Haraldsdottir et al. 2020) that is important for engagement, clinical development and professional growth (Peet et al. 2022). A requirement for the creation of reflective spaces that are safe (Dickinson et al. 2005; Dickinson et al. 2008) neutral (Moreno‐Poyato et al. 2019), formalised and protected (Moreno‐Poyato et al. 2019; Haraldsdottir et al. 2020) and facilitate exploration of staff's own feelings and experiences (Bagaragaza et al. 2024; Moreno‐Poyato et al. 2019; Noergaard et al. 2016; Haraldsdottir et al. 2025) is highlighted.

Staff value opportunities to connect and collaborate meaningfully with other staff (Peet et al. 2022; Jackson et al. 2025; Haraldsdottir et al. 2020; Venturato et al. 2020; Haraldsdottir et al. 2025) and highlight the benefit of gaining multiple views and perspectives on practice issues through engaging in PD (Haraldsdottir et al. 2020; Venturato et al. 2020). PD discussions facilitated in a safe, inclusive and open environment may have a positive effect on team dynamics (McCance et al. 2013; Laird et al. 2015; Bagaragaza et al. 2024; Dickinson et al. 2005; Dickinson et al. 2008). Opportunities for connection and collaboration through PD foster respect and good relationships between staff, which is subsequently beneficial for patient experiences and outcomes (McCance et al. 2013; Laird et al. 2015; Dickson et al. 2018; McCormack et al. 2018; Donegan et al. 2021; Peet et al. 2022; Haraldsdottir et al. 2020). Several included papers demonstrate how improved collaboration and communication fostered through PD are not confined to staff members alone but also yield significant benefits in interactions with service users, relatives and families (Boomer et al. 2019; Dempsey 2008; Drayton et al. 2021; Brown and McCormack 2011; Armitage and Evans 2005; Noergaard et al. 2016). This may facilitate family input into care, which is ultimately beneficial for staff and patient experiences (Boomer et al. 2019; Dempsey 2008; Drayton et al. 2021). Opportunities to reflect on relatives' experiences facilitated both recognition and active support of the needs of families whilst their relatives were receiving care (Boomer et al. 2019; Drayton et al. 2021; Armitage and Evans 2005; Noergaard et al. 2016).

Staff perceive the relevance of PD activities as “most relevant [and] related to our daily working life challenges” (Dahl et al. 2018). PD and change initiatives that are underpinned by collaborative engagement with all relevant staff and teams contributing to the specific area of practice are more likely to yield outcomes perceived as meaningful and responsive to the needs of both healthcare professionals and patients (Dickson et al. 2018; McCormack et al. 2018; Dahl et al. 2018; Noergaard et al. 2016; Haraldsdottir et al. 2020). However, barriers such as individuals perceived comfort and willingness to communicate in group settings (Dahl et al. 2018) negative attitudes and behaviours (Dickson et al. 2018; McCormack et al. 2018) and one‐way communication practices (McCance et al. 2013; Laird et al. 2015) may limit PD collaboration.

Engagement in PD may increase the professional and clinical confidence of nursing staff (Miskelly and Duncan 2014; Moreno‐Poyato et al. 2019; Peet et al. 2022), helping nurses to recognise the validity and importance of the nursing voice (Peet et al. 2022) and develop underpinning clinical and professional knowledge (Miskelly and Duncan 2014). Staff report enjoyment of PD activities and see the value of their engagement with each‐other and with the PD process (McCance et al. 2013; Laird et al. 2015; Jackson et al. 2025; Haraldsdottir et al. 2020), and the outputs of PD (Bellman et al. 2003). Through engaging with PD, staff not only perceive their own meaningful engagement, but also that of others (Bellman et al. 2003; Moreno‐Poyato et al. 2019) which may increase motivation amongst teams (Miskelly and Duncan 2014; Moreno‐Poyato et al. 2019). Collaborative approaches that underpin PD may also assist in enhancing communication across management hierarchies (Miskelly and Duncan 2014; Brown and McCormack 2011; Afshar et al. 2020), serving as a “great way to break down barriers and develop a deeper understanding of why some decisions are made and to take time to look at the bigger picture” (Miskelly and Duncan 2014).

##### 3.4.1.5 Identification of Workforce Needs

The PD process appears to inherently reveal staff learning and development needs required for PD (Moreno‐Poyato et al. 2019; Peet et al. 2022; Armitage and Evans 2005). In some cases, this may enable development of bespoke training and education programmes to bridge specific knowledge gaps within the workforce (Miskelly and Duncan 2014; Bagaragaza et al. 2024; Donegan et al. 2021; Blanchfield and O'Connor 2022; Haraldsdottir et al. 2025), which may offer opportunities to tailor educational interventions to better meet the combined needs of patients and staff.

The facilitative approaches often adopted by PD were felt to better enable collaboration compared with didactic approaches (McCance et al. 2013; Laird et al. 2015; Dickson et al. 2018; McCormack et al. 2018; Bagaragaza et al. 2024). Indeed, the active learning approaches utilised in PD may be seen as a new way of learning, which may be further enhanced through action research approaches (Bellman et al. 2003; Friesen‐Storms et al. 2015; Venturato et al. 2020). Furthermore, these training and education opportunities offered through PD may be a stimulus for individual professional aspiration such as further study (Miskelly and Duncan 2014; Peet et al. 2022) or seeking publication (Bellman et al. 2003).

Some barriers to training and education are noted, including assessment tools being perceived as too complicated to use in practice (Dempsey 2008), low utilisation of recommended websites for training (Friesen‐Storms et al. 2015), short duration of facilitation sessions (Dahl et al. 2018) and achieving consistency in the knowledge and skills of PD facilitators (McCance et al. 2013; Laird et al. 2015).

#### 3.4.2 Theme 2: Enablers and Constraints in Practice Development

##### 3.4.2.1 Values, Attitudes and Environments

The collaborative nature of PD facilitates opportunities for discussion and reflection amongst staff that may not otherwise occur organically. These opportunities appear to provide a chance for human‐centred, action orientated decision making based on shared values and consensus (Bagaragaza et al. 2024; Moreno‐Poyato et al. 2019; Peet et al. 2022; Drayton et al. 2021; Jackson et al. 2025; Dickinson et al. 2005; Dickinson et al. 2008; Haraldsdottir et al. 2025). Across the included papers, person‐centeredness appears as key feature of successful PD initiatives. The concepts of knowing the patient as a person (Drayton et al. 2021; Dickinson et al. 2005; Dickinson et al. 2008), making responsive practice changes to meet patient's individual needs (Drayton et al. 2021; Brown and McCormack 2011; Dickinson et al. 2005; Dickinson et al. 2008), involving patients as collaborators and contributors to their care (Moreno‐Poyato et al. 2019; Drayton et al. 2021) and higher‐quality interactions between staff and patients/families (Boomer et al. 2019; Drayton et al. 2021; Noergaard et al. 2016; Skene et al. 2019; Venturato et al. 2020) appear to be key facilitators for the enhancement of person‐centredness.

The positive attitudes of staff towards PD as a mechanism for practice change and improvement facilitate success and impact (McCance et al. 2013; Laird et al. 2015; Dickson et al. 2018; McCormack et al. 2018). In some cases, engaging with the PD process may alter individual staff attitudes towards care, including increased recognition of emotional care (Boomer et al. 2019) and recognition of aspects of practice that require change (McCance et al. 2013; Laird et al. 2015). Whilst positive staff attitudes may facilitate PD success, external factors such as time constraints (Friesen‐Storms et al. 2015; Drayton et al. 2021; Blanchfield and O'Connor 2022) and service pressures (McCance et al. 2013; Laird et al. 2015; Brown and McCormack 2011) may undermine positive attitudes and act as significant barriers. Participation in PD, and related learning opportunities, can improve the clinical and professional abilities of staff (Bellman et al. 2003; Moreno‐Poyato et al. 2019; Friesen‐Storms et al. 2015). Following PD, staff may perceive improved ways of working, with increased productivity and effectiveness (Bellman et al. 2003; Moreno‐Poyato et al. 2019). However, the potential for scepticism (Bellman et al. 2003), power struggles (McCance et al. 2013; Laird et al. 2015; Friesen‐Storms et al. 2015) and expectation of unpaid attendance (Venturato et al. 2020) may negatively impact PD success. Furthermore, PD interventions may not provide “blanket improvements”, and flexibility and sensitivity to individual situations and contexts may still be required (Dahl et al. 2018).

##### 3.4.2.2 Technology

Despite heavy reliance on data and technology interfaces in modern healthcare practice, there appears to be a lack of technology integration or exploration in the included studies. Although technology may act as a barrier to PD (Friesen‐Storms et al. 2015) this is sparsely addressed in the papers retrieved, suggesting a gap in knowledge and understanding of how technology may facilitate or hinder PD:

Discharge registration has become quite difficult. We must open a lot of additional windows and enter a lot of ticks. In some instances it is not clear to me where I should find the right window and what I need to tick. In view of the time I need to find the right windows, I do not register at all. (Friesen‐Storms et al. 2015)

One study (Friesen‐Storms et al. 2015) included an IT specialist as part of the PD group (as a participating co‐researcher); however, this was not evident in any other included study. Regardless of rapid increases in the use and integration of technology in healthcare over recent years, a general lack of technology integration in PD was evident across the timespan of included studies, including those more recently published.

##### 3.4.2.3 Staff Experience, Satisfaction and Safety

Engaging in PD may help staff reconnect with aspects of their work that they find meaningful, worthwhile and rewarding (McCance et al. 2013; Laird et al. 2015; Peet et al. 2022; Brown and McCormack 2011; Jackson et al. 2025; Haraldsdottir et al. 2020), and may enhance morale (Bagaragaza et al. 2024; Brown and McCormack 2011) and professional identity (McCance et al. 2013; Laird et al. 2015; Peet et al. 2022). PD encourages staff to engage in self‐reflection, considering their own capabilities, skills and practice (McCance et al. 2013; Laird et al. 2015; Ling (Iris) Li et al. 2010). Staff gained confidence to step out of comfort zones (Venturato et al. 2020), and to use their enhanced self‐awareness to generate improvements (Moreno‐Poyato et al. 2019). Following the PD intervention, staff reported increases in satisfaction (Venturato et al. 2020), happiness and optimism (Venturato et al. 2020), and increased enjoyment in work (Dickinson et al. 2005; Dickinson et al. 2008). As PD offers the opportunity to gain multiple perspectives, potential unintended consequences may be avoided prior to implementing change (Brown and McCormack 2011). Staff also perceive that practice changes made as a result of PD may improve care processes (Drayton et al. 2021), increase long‐term efficiency, support respectful care (Dahl et al. 2018) and enhance person centredness (Drayton et al. 2021). As a result of participating in PD, staff experienced increased confidence and amplified voice (Peet et al. 2022) whilst also working to equalise power‐balance with patients and families (Peet et al. 2022; Skene et al. 2019).

One paper included a staff reflection on how changes that occurred as a result of PD had a positive impact on staff stress due to more efficient nursing processes (Dickinson et al. 2005; Dickinson et al. 2008). In another, a staff member's perception of their own safety was enhanced as a result of changes made through PD:

When I see my colleagues doing a bedside GCS it empowers me to be really thorough in my assessment. […] I feel safer on the ward knowing we are doing things like that. (Peet et al. 2022).

Interestingly, whilst there is evidence that engaging in PD enhances staff satisfaction, explicit attention or investigation of the impact of PD on staff wellbeing appears to be somewhat minimal. Nurses may perceive increased recognition (Peet et al. 2022) and respect (Afshar et al. 2020; Haraldsdottir et al. 2020) of their role as a result of PD activity. Two papers highlight that in enhancing awareness of person‐centredness, staff had also realised that the principle “*applies to them too, not just the patients*” (McCance et al. 2013; Laird et al. 2015; Dickson et al. 2018; McCormack et al. 2018). However, despite acknowledging the impact of low or short staffing levels and high workload pressures, few papers discussed the impacts of PD initiatives on staff burnout. In the three papers that indirectly addressed this, staff highlighted how their own wellbeing could be overshadowed during periods of high work pressures, leading feelings of not being able to cope, working too fast, or not fast enough (Dickinson et al. 2005; Dickinson et al. 2008; Haraldsdottir et al. 2020), or feelings of exhaustion (Dahl et al. 2018). In two papers, staff made links between their own experiences and feelings associated with burnout, and the subsequent negative impacts on patient care (Dahl et al. 2018; Haraldsdottir et al. 2020).

##### 3.4.2.4 Team Dynamics

Engagement with and within teams appears to be a key facilitator for the success of PD. Staff report valuing opportunities to come together as a team (Bagaragaza et al. 2024; Peet et al. 2022; Venturato et al. 2020), and how this increases perceptions of support (Brown and McCormack 2011). Respect and relationship improvements across professional hierarchies, for example between nurses and care assistants (Boomer et al. 2019) and general practitioners (GP's) and practice nurses are evident. Overall, opportunities to work together as a wider team may lead to better understanding of the wider system on practices at meso and micro levels (Miskelly and Duncan 2014) and better understanding of the roles, knowledge, and experiences of other team members (Boomer et al. 2019). This can strengthen relationships across wider teams (Dickson et al. 2018; McCormack et al. 2018) and cultivate a sense of mutual respect (Haraldsdottir et al. 2020), which is further enhanced by fostering deeper interpersonal connections with colleagues (Dickson et al. 2018; McCormack et al. 2018). Team communication was enhanced at individual (Brown and McCormack 2011) and team level, with discussions taking place in safe, open, and honest ways positively impacting on team cohesion (Dempsey 2008; Moreno‐Poyato et al. 2019; Armitage and Evans 2005; Afshar et al. 2020).

##### 3.4.2.5 Patient Involvement

Across the included qualitative studies, approaches to patient involvement in PD were varied and inconsistent and sometimes multi‐layered in approach. Methods of patient involvement varied from examination of patient data (Donegan et al. 2021; Friesen‐Storms et al. 2015; Drayton et al. 2021) to interviews or focus groups with patients and/or carers (McCance et al. 2013; Laird et al. 2015; Dahl et al. 2018; Dempsey 2008; Friesen‐Storms et al. 2015; Drayton et al. 2021; Blanchfield and O'Connor 2022; Armitage and Evans 2005; Noergaard et al. 2016; Skene et al. 2019), observation (Dahl et al. 2018; Noergaard et al. 2016; Skene et al. 2019; Dickinson et al. 2005; Dickinson et al. 2008; Haraldsdottir et al. 2020), patient and carer surveys (Donegan et al. 2021; Drayton et al. 2021; Afshar et al. 2020), parent diaries (Skene et al. 2019), and in some cases, patient involvement in PD teams (Skene et al. 2019; Haraldsdottir et al. 2020). Reporting of patient involvement was also inconsistent. In some cases, the data gathered from patients was done so formally, using qualitative research methods (McCance et al. 2013; Laird et al. 2015; Dahl et al. 2018; Friesen‐Storms et al. 2015; Drayton et al. 2021; Brown and McCormack 2011; Blanchfield and O'Connor 2022; Armitage and Evans 2005; Noergaard et al. 2016; Skene et al. 2019; Dickinson et al. 2005; Dickinson et al. 2008), and in others, patient involvement was reported informally, or through the perceptions of staff/PD team members (Dahl et al. 2018; Haraldsdottir et al. 2020). Owing to wide variations and inconsistent reporting of qualitative data, it is difficult to conclude the effect of patient involvement on the impact, success and sustainability of PD within this systematic review.

#### 3.4.3 Theme 3: Collective Empowerment

##### 3.4.3.1 Empowerment and Emancipation

Emancipation of nursing staff is characterised at an individual level as personal and professional growth (Boomer et al. 2019; Miskelly and Duncan 2014; Bellman et al. 2003; Moreno‐Poyato et al. 2019), increased confidence (Miskelly and Duncan 2014; Bagaragaza et al. 2024; Peet et al. 2022), professional aspiration (Miskelly and Duncan 2014; Bellman et al. 2003) self‐efficacy (Miskelly and Duncan 2014; Brown and McCormack 2011) and perceived ability to affect change (Moreno‐Poyato et al. 2019; Armitage and Evans 2005). This emancipation may in turn facilitate and support successful generation of cultures of empowerment (Boomer et al. 2019; Brown and McCormack 2011; Venturato et al. 2020). Through engaging with PD Staff may be empowered to challenge and change hierarchical structures (Boomer et al. 2019; Bellman et al. 2003; Peet et al. 2022; Brown and McCormack 2011; Haraldsdottir et al. 2020), thus engaging in a process of emancipation. The flexibility of the PD approach may further empower staff to participate in PD as implementation strategies can be adapted and tailored, giving staff voice in decision‐making (Friesen‐Storms et al. 2015). However, one study highlights the potential risks associated with emancipation and empowerment of staff, and the importance of ongoing commitment to maintaining a culture of empowerment.

You don't empower people and then disempower them…in some ways it's very disconcerting and quite ruthless to do (Bellman et al. 2003)

Additionally, whilst the concept of empowering the workforce to generate change “from the bottom up” is recognised as potentially “frightening for some” (Bellman et al. 2003), this may indeed validate the ability of PD to emancipate nursing staff by empowering them to challenge existing hierarchies and perceptions of power.

##### 3.4.3.2 Leadership

The role and impact of leadership on the success, impact and process of PD was inconsistently addressed in the included papers. In those that did acknowledge the role of leadership in PD, there was evidence of behavioural approaches such as role modelling being utilised by leaders and managers to support staff to implement PD interventions (McCance et al. 2013; Laird et al. 2015; Dickson et al. 2018; McCormack et al. 2018; Brown and McCormack 2011). In addition to role modelling clinical skills such as ward rounds (Brown and McCormack 2011), role modelling values and behaviours is also noted as being a key factor in successfully engaging clinical staff (Dickson et al. 2018; McCormack et al. 2018). The visibility of leaders and managers may enhance communication across pre‐existing hierarchies (Venturato et al. 2020), and conversely, lack of leadership presence in PD activities may hinder opportunities for problem solving and action for change (Moreno‐Poyato et al. 2019). One paper noted that the impact of PD discussions was limited by the absence of the unit supervisor, hindering consensus and implementation of solutions (Moreno‐Poyato et al. 2019). A disconnect may also occur between organisational aims and management practices, with values such as person‐centredness felt to not be valued by managers as may be perceived as conflicting with other priorities (Dickson et al. 2018; McCormack et al. 2018). Consistency in management support may be required at all organisational levels, as one paper highlights how a local merger caused structural destabilisation that impacted the PD study:

Phase one of the study has been undertaken within an organisational climate of competing priorities, the resignation of the Director of Nursing, and a local merger with another NHS hospital. These structural influences can destabilise the workplace and certainly impacted on the study. (Bellman et al. 2003).

In addition to being required for the successful implementation of PD, leadership in PD also appears to have an emancipatory function. Where staff perceive that they are valued and supported by managers and leaders, they experience a sense of empowerment to embrace opportunities for developing practice (Miskelly and Duncan 2014; Bellman et al. 2003; Venturato et al. 2020) and developing their own leadership skills (Miskelly and Duncan 2014). Alongside emancipation, PD approaches may flatten existing hierarchy and authority gradients, enabling staff to design increased opportunities for meetings with leadership (Haraldsdottir et al. 2020) and to distribute power between senior and junior staff (Brown and McCormack 2011).

##### 3.4.3.3 Patient Empowerment

The inclusion of patients in PD serves to amplify patient voice, foster empowerment, and facilitate meaningful engagement in the co‐construction of care practices that directly influence patient experiences and outcomes. Many PD exploratory activities highlighted that both staff and patients simultaneously felt the impact of suboptimal conditions, care processes or constraints (McCance et al. 2013; Laird et al. 2015; Dempsey 2008; Drayton et al. 2021; Skene et al. 2019). Developing these areas of practice may increase patient satisfaction and experiences as a result of PD; improvements are evident in improved relational quality between patients and staff resulting from enhanced person‐centredness (Donegan et al. 2021; Drayton et al. 2021; Haraldsdottir et al. 2020), therapeutic relationships (Moreno‐Poyato et al. 2019; Skene et al. 2019), responsiveness (McCance et al. 2013; Laird et al. 2015; Dempsey 2008; Drayton et al. 2021) and trust (Moreno‐Poyato et al. 2019; Haraldsdottir et al. 2020; Ling (Iris) Li et al. 2010). Beneficial impacts such as enhanced HCP‐patient relational quality and increased service efficiency (Armitage and Evans 2005) are also perceived by staff. The included papers illustrate the ability of PD to increase patient and family inclusion and empowerment (Boomer et al. 2019; Drayton et al. 2021; Armitage and Evans 2005; Skene et al. 2019) In some cases, these enhancements were underpinned by practice changes, such as extending the content of family conferences to include the role of families in providing care, and discussion/referral of family support needs in weekly multidisciplinary team meetings (Armitage and Evans 2005). This enhanced collaboration with relatives and families may enhance individualised care, as input from the family provides valuable information regarding individual needs and preferences (Dempsey 2008; Drayton et al. 2021). Similarly, in NICU settings, enhanced inclusion and collaboration with parents was found to empower parents to participate in caregiving and learning about neonatal behaviours (Noergaard et al. 2016; Skene et al. 2019).

### 3.5 Quantitative Findings

The types and measures of quantitative outcomes collected by the two quantitative and 12 mixed method studies differed across the included papers. Two mixed methods design studies (Bogerd et al. 2024; Jackson et al. 2025) did not include their quantitative data and therefore are not included in this quantitative analysis. Quantitative data was therefore extracted from 13 studies. Number and frequency were the most commonly reported measures, followed by means with and without their standard deviations. In the included quantitative data, there were limited statistical tests conducted to compare values: *t*‐tests (Afshar et al. 2020; Skene et al. 2019; Heshmatifar et al. 2025), Mc Nemars test (Hestevik et al. 2019), change score (Blanchfield and O'Connor 2022) and logistic models (Bucknall et al. 2022). It is therefore not possible to present summary descriptive statistics. Instead, a narrative overview of key quantitative findings is presented.

Quantitative outcomes were most commonly captured using routinely collected data (Boomer et al. 2019; Dickson et al. 2018; McCormack et al. 2018; Bucknall et al. 2022; Dempsey 2008; Donegan et al. 2021; Hestevik et al. 2019; Blanchfield and O'Connor 2022; Armitage and Evans 2005) followed by questionnaires (Miskelly and Duncan 2014; Dempsey 2008; Donegan et al. 2021; Drayton et al. 2021; Brown and McCormack 2011; Afshar et al. 2020; Skene et al. 2019; Heshmatifar et al. 2025).

Family and patient involvement was the most common reported outcome. Increased communication with patients (Boomer et al. 2019) and family members (Boomer et al. 2019; Armitage and Evans 2005) on end‐of‐life care; improved joint decision making with patients (Drayton et al. 2021) or family members (Skene et al. 2019). Other positive outcomes following implementation of PD included greater patient and job satisfaction for nurses (McCormack et al. 2018; Afshar et al. 2020) and increased use of library resources by nurses (Miskelly and Duncan 2014). Due to inconsistencies in measuring and reporting short and long‐term impacts of PD upon culture change, this outcome is difficult to ascertain with certainty.

Two studies captured clinical measurements; patients with type 2 diabetes and chronic kidney disease who received care from an advanced nurse practitioner (ANP) (Blanchfield and O'Connor 2022) and nurses' vital sign measurement, interpretation, treatment and escalation of care (Bucknall et al. 2022). PD, for patients attending combined ANP care, had minimal or no impact on lifestyle factors (i.e., smoking and exercise). For clinical outcomes, the greatest impact was a reduction of 24% in low transferrin saturation followed by a 23.5% decrease in low haemoglobin. PD had no or minimal impact on blood pressure, cholesterol or body mass index. However, non‐attendance for review was the lowest for ANP care compared to physician‐led services. PD did initially improve escalation of patient care, but this impact was not sustained. Although PD did not change the rate of unplanned ICU admission, mortality was reduced in the intervention group. Reduction in mean length of in‐patient hospital stay was reported by two studies; seven (Donegan et al. 2021) and two (Bucknall et al. 2022) days, respectively for patients who on average were in their sixties or older.

One study reported mixed results. A Norwegian study (Hestevik et al. 2019) on nutritional care documentation in a nursing home reported higher rates of elderly patients at nutritional risk or severely malnourished at follow‐up (increase of 50%). It is not established if the PD intervention influenced this result by increasing awareness of nutritional practice guidelines. However, if this was true then it would not explain why the study also reported a decrease in the amount of residents who had a recorded weight (decrease of 21.2%) and nutritional status assessment (decrease of 29.5%). The study also reported an increase in the proportion of documentation on dietary intake (increase of 8.4%) and requirement calculations (increase of 2.5%).

Clinical outcomes were inconsistently and scarcely reported across the included papers. This may be because most papers used qualitative data collection methods, which somewhat limits the ability to measure clinical outcomes over periods of time. Where clinical outcomes were addressed qualitatively, involvement of patients in their treatment goals (21), completions of nursing care plans (16) and nurse‐led medication audit of adverse medication effects (8) are noted to be improved as a result of PD interventions. Overall, a lack of quantitative measurement or reporting of clinical outcomes occurring as a result of PD makes it difficult to draw conclusions from the included papers. Interestingly, none of the included studies explicitly measured or reported the impact of PD on patient quality of life (QOL) despite the existence of validated QOL measurement tools.

## 4 Discussion

This review primarily aimed to explore the existing literature on PD interventions, with and without PAR, involving patient and/or staff and reporting on outcomes and/or experiences. Contextual factors associated with PD intervention and their impact on patient and/or staff outcomes and/or experiences were considered as a secondary aim, alongside investigation of the relationship between PD intervention characteristics and patient and/or staff outcomes and/or experiences.

The findings from the majority of studies suggest that PD, over different study designs, appears to benefit the environment in which staff work and patients are cared for. However, PD does not currently show a consistent benefit for clinical outcomes or quality of life. A central explanation for this inconsistency is the nature of PD itself. PD is not a standardised intervention deployed in a uniform way; rather, it is a flexible and adaptive approach shaped by local needs, values, resources and contexts. This flexibility is a defining strength of PD, enabling responsiveness and relevance, but it also complicates validation, comparison and synthesis across studies. PD interventions therefore cannot be compared like‐for‐like, and expectations of consistent clinical outcomes may not align with PD's primary purpose.

The review also highlights persistent ambiguity in how PD activity is positioned as research or quality improvement. This is demonstrated through the conflict some authors had defining if their use of PD was a vehicle for quality improvement or research. Indeed, some of the papers excluded at the screening stage outlined how ethical approval was sought only to be informed that their activity was not considered research (Pontivivo et al. 2012; Hennessey and Fry 2016; Harvey and Cameron 2021). Reaching a consensus on which papers were research versus quality improvement resulted in rich methodological discussions amongst the review team. These tensions reflect broader debates about knowledge generation versus knowledge application. Quality improvement is primarily concerned with using existing knowledge to improve care, services and work processes, whereas research focuses on generating new information. PD spans this boundary; it can generate new knowledge while simultaneously improving practice. Several PD principles identified in this review, person‐centredness, values clarification, facilitation and cultural change, may strengthen quality improvement approaches by embedding reflection, participation and sustainability into improvement efforts. Rather than positioning PD in opposition to quality improvement, future work could explicitly describe how PD principles are operationalised within quality improvement initiatives, and where PD extends beyond improvement into knowledge generation. We suggest that this is an important gap to address as the value of, and need for, PD grows in increasingly complex modern healthcare systems.

This review identified that, where PD is employed for purposes of research, there is a lack of rigour in conduct and reporting. A small number of the included qualitative studies explicitly addressed rigour (Bagaragaza et al. 2024; Friesen‐Storms et al. 2015; Blanchfield and O'Connor 2022; Haraldsdottir et al. 2025). There also appears to be an absence of standardised reporting guidelines associated with PD methodologies. The importance of improving rigour becomes more pronounced where PAR methods are employed alongside PD. This combination was frequently observed in the included papers, with the most commonly observed combination being PD and PAR (Dahl et al. 2018; Hestevik et al. 2019; Moreno‐Poyato et al. 2019; Friesen‐Storms et al. 2015; Blanchfield and O'Connor 2022; Afshar et al. 2020; Noergaard et al. 2016; Skene et al. 2019; Zaforteza et al. 2015; Jackson et al. 2025; Ling (Iris) Li et al. 2010; Venturato et al. 2020). Other notable combinations included PD with Emancipatory Action Research (Brown and McCormack 2011; Dickinson et al. 2005; Dickinson et al. 2008), Appreciative Inquiry (Drayton et al. 2021), and Critical Action Research (Bellman et al. 2003). Additionally, some studies combined PD with Critical Ethnography (Dahl et al. 2018) or employed Emancipatory PD (Peet et al. 2022). A cyclical process, a core principle of PD, was evident in the majority of included studies. Notably, all studies that combined PD and PAR demonstrated cyclical processes. In contrast, amongst the 17 studies that employed PD without PAR, five did not exhibit cyclical processes, suggesting that the integration of PAR may support the enactment of this key PD principle. Clarity about which aspects of PD are fixed and which are flexible may help advance the science of PD while preserving contextual responsiveness.

PD itself is a continuous process adapted to suit the aims, needs and resources of each setting. However, this review highlighted that, broadly, studies did not explicitly acknowledge their assumptions of PD or of their research paradigm, and in the absence of this, whether the PD project was in fact quality and/or service improvement. In several papers, information on methods of recruitment or study population characteristics (including eligibility criteria) was limited or absent. This made it very difficult to examine the applicability and acceptability of PD in healthcare settings which care for patients and their families with different needs at different points in their life.

Overall, a significant proportion of the papers retrieved had a central tenet of person‐centredness (McCance et al. 2013; Laird et al. 2015; Dickson et al. 2018; McCormack et al. 2018; Dempsey 2008; Donegan et al. 2021; Drayton et al. 2021; Haraldsdottir et al. 2020). More widely, McCormack et al. describe “person‐centredness” as:

an approach to practice established through the formation and fostering of healthful relationships between all care providers, service users and others significant to them in their lives. It is underpinned by values of respect for persons, individual right to self‐determination, mutual respect and understanding. It is enabled by cultures of empowerment that foster continuous approaches to Practice Development. (Riddett 2017)

Interestingly, this review identified that PD had facilitated healthcare staff in acknowledging that the principle of person‐centeredness applied to themselves as well as to patients (McCance et al. 2013; Laird et al. 2015; Dickson et al. 2018; McCormack et al. 2018). This finding is potentially impactful for supporting wider patient safety agendas and systems approaches, which advocate that staff wellbeing is intrinsically interconnected with patient safety and both can be influenced by the design (and redesign) of work systems (Carayon et al. 2006). Poor staff wellbeing and high levels of burnout are associated with poor patient safety outcomes (Hall et al. 2016) and depleted staffing (Kirk 2024). In this review, no studies explicitly addressed the potential for PD to support staff wellbeing or address burnout, however this absence may suggest that this may be a future area for exploration.

Although many papers highlighted the aim of PD as addressing power structures and empowering staff as co‐researchers, there was limited clarity in the definition and utilisation of ‘co‐researchers’. It is not always clear how co‐researchers are embedded within a study, what their overall contribution is, and how power structures were ultimately addressed or changed. No study stated utilisation of agreed definitions or guidelines for co‐researchers, and it is unclear whether such consensus exists. We suggest that increasing clarity in this area has the potential to increase transparency and potentially the quality of PD.

This review finds evidence that PD may help to create working conditions where existing hierarchy is flattened and authority gradients are reduced. This finding has significance for patient safety, as flatter power hierarchies are evidenced to support safety behaviours such as speaking up (Kim et al. 2020; Mawuena and Wilkinson 2024). This review reveals that reticence to relinquish power or empower others may present a barrier to PD; however, it may also be that PD itself provides an opportunity to explore perceptions of power, hierarchy and its impact on patient safety. It may be that PD, being participatory in nature and demonstrating the ability to enhance communication and collaboration between wider teams, may be well placed to examine this phenomenon further. Given these findings, it is possible to propose the core components of an ‘ideal’ PD intervention for future testing, while recognising that such an intervention must remain adaptable. These components include: explicit articulation of values and purpose; use of cyclical and reflective processes; meaningful practitioner involvement in knowledge generation; organisational support for cultural change; and transparent reporting of theory, assumptions and context.

Remarkably, none of the included studies explicitly measured or reported the impact of PD on patient quality of life, although it could be argued that this was implied in some studies. This may be due to the deeply qualitative nature of this phenomenon, despite the existence of validated quality of life measurement tools. This review highlights a knowledge gap in this area which, particularly in areas such as palliative care, could be beneficial to address. It is also important to note that only one hospital setting was based in a psychiatric unit (Moreno‐Poyato et al. 2019), suggesting a potential under‐use of PD in mental health settings. However, whilst this systematic review adds to the body of knowledge, we have shown that the majority of research on PD in healthcare settings is undertaken in English speaking countries and countries that are predominantly Caucasian. As most studies examined work‐based and not person‐based cultural competencies, it is unclear how well PD would transfer to other settings.

This review finds some evidence that PD is associated with positive outcomes, but few findings showed a clinical benefit. This should be considered both when designing and evaluating PD. Currently, it would broadly appear that PD may be best suited to improve work‐based and patient environment(s).

## 5 Strengths and Limitations

This review demonstrates several methodological strengths. The evidence‐based for PD was examined utilising both grey and peer reviewed publications, and a comprehensive systematic search strategy was employed, encompassing multiple databases to ensure broad and inclusive coverage of relevant literature. The search period was deliberately extended beyond the conventional ten‐year limit, spanning from 1st January 1980 to 5th January 2026, in recognition of a perceived paucity of literature directly addressing the scope and aims of this review. While this inclusive approach facilitated the inclusion of seminal studies, some earlier publications presented methodological limitations, which introduced certain constraints to the overall quality of the evidence base. The intersection between quality improvement and PD resulted in rich methodological discussions; however, the interpretive nature of these debates alongside the absence of wider guidance or differentiation between quality improvement and PD may be perceived as an inherent limitation.

Limiting the search to the English language may introduce an element of bias. The search strategy was robust to avoid lower recall rates. By including search terms within the Comparator(s) and Outcome(s) across various databases we prioritised the population (patients and staff) and the intervention (PD). We ran a test pilot of our search strategy to ensure that as many articles as possible would be retrieved. We recognised that PD could exist in other settings under a different term and ensured our review had a definition for PD to ensure inclusivity when reviewing the literature. However, we are mindful that our reliance on specific terminology may have inadvertently missed some relevant publications while conducting the search strategy. As PD represents a transformative process oriented towards person‐centred care, organisational culture, and broader systemic influences, authors may have employed alternative terminology to describe similar phenomena.

PRISMA guidelines were adhered to. Dual independent screening and data extraction were conducted using pre‐designed templates, and specialist review software was used to manage screening and extraction. Risk of bias was systematically assessed using the Mixed Methods Appraisal Tool (MMAT) independently by multiple reviewers. Furthermore, advanced synthesis techniques were utilised, which enabled the integration of contextual insights into the analysis.

Data limitations included heterogeneity in study designs and small sample sizes. Additionally, several retrieved papers were excluded on the basis that they were service evaluations rather than empirical research studies. A key strength of the included studies using single design was in their methodological approach—as demonstrated by the MMAT assessments. The overall limitations of the studies were the fluidity of both terminology and reporting approach to research studies. The generalisability of findings may also be constrained by contextual factors such as varying levels of familiarity with PD methodologies, varying access to resources, and the limited sustainability of interventions beyond the duration of funded projects. Finally, the scope of the review excluded maternity care, which may restrict the transferability of its findings to this setting.

## 6 Conclusion

Evidence suggests that PD contributes positively to the working environment for staff and the care environment for patients. However, the current body of research does not demonstrate consistent improvements in clinical outcomes or quality of life. PD is inherently labour and resource intensive due to a reliance on engagement, participation and democratic exploration of complex healthcare work related issues. Its successful implementation and long‐term sustainability are contingent upon robust organisational support, particularly from leadership and management across all organisational levels. In the current climate of heightened service demand and constrained capacity, the complex and dynamic nature of PD presents both challenges and great opportunities to potentially enhance staff and patient experiences, and to improve the conditions of healthcare environments for all.

## 7 Impact and Implications for Practice

This review has highlighted how PD in healthcare, primarily hospital settings, may work to improve the work and care environment for staff and patients, respectively. Additionally, this review reveals gaps in PD knowledge that would be beneficial to explore: these include the use of PD in mental health settings, broadening definitions of cultural competency, greater inclusion of healthcare staff beyond nursing, and investigation of PD in relation to clinical outcomes. Consistency in applying PD principles and reporting PD studies may be supported by the development of standardised practices and reporting guidelines, improving the overall rigour of the PD methodology.

## Funding

The THEO project is funded by Legacy Health Education England through the Norfolk Initiative for Coastal and Rural Health Equalities (NICHE), hosted by the University of East Anglia. Please see https://www.uea.ac.uk/groups‐and‐centres/projects/niche for more information.

## Conflicts of Interest

All named authors formed part of the THErapeutic Optimisation for Effective Integrated Care Delivery (THEO) Project. This systematic review is part of the THEO study and supports the development of the Study intervention, which comprises a framework of Practice Development activities and an enhanced staffing model.

## Data Availability

The data that support the findings of this study are available from the corresponding author upon reasonable request.
